# Characterization of pertussis-like toxin from *Salmonella* spp. that catalyzes ADP-ribosylation of G proteins

**DOI:** 10.1038/s41598-017-02517-2

**Published:** 2017-06-01

**Authors:** Yukino Tamamura, Kiyoshi Tanaka, Ikuo Uchida

**Affiliations:** 1 Bacterial and Parasitic Diseases Research Division, National Institute of Animal Health, Tsukuba, Ibaraki 305-0856 Japan; 2 Dairy Hygiene Research Division, Hokkaido Research Station, National Institute of Animal Health, 4 Hitsujigaoka, Toyohira, Sapporo 062-0045 Japan; 3 Veterinary Bacteriology, Department of Pathology, School of Veterinary Medicine, Rakuno Gakuen University, 582 Bunkyoudai-midorimachi, Ebetsu, Hokkaido 069-8501 Japan

## Abstract

*Salmonella* Typhimurium definitive phage type (DT) 104 produces a pertussis-like toxin (ArtAB-DT104), which catalyzes ADP-ribosylation of pertussis toxin sensitive G proteins. However, the prevalence of ArtAB and its toxicity have not been established. We report here that, in addition to DT104, *S*. Worthington, and *S*. *bongori*, produce ArtAB homologs, designated ArtAB-SW and ArtAB-Sb, respectively. We purified and characterized these ArtAB toxins, which comprise a 27-kDa A subunit (ArtA) and 13.8-kDa pentameric B subunits (ArtB). While the sequence of the A subunit, which is ADP-ribosyltransferase, is similar to the A subunit sequences of other ArtABs, the B subunit of ArtAB-Sb is divergent compared to the B subunit sequences of other ArtABs. Intraperitoneal injection of purified ArtABs was fatal in mice; the 50% lethal doses of ArtAB-DT104 and ArtAB-SW were lower than that of ArtAB-Sb, suggesting that ArtB plays an influential role in the toxicity of ArtABs. ArtABs catalyzed ADP-ribosylation of G proteins in RAW 264.7 murine macrophage-like cells, and increased intracellular cyclic AMP levels. ArtAB-DT104 and ArtAB-SW, but not ArtAB-Sb, stimulated insulin secretion in mice; however, unlike Ptx, ArtABs did not induce leukocytosis. This disparity in biological activity may be explained by differences in ADP-ribosylation of target G proteins.

## Introduction

*Salmonella* spp. cause infectious diseases, such as gastroenteritis, which is associated with diarrhea and inflammatory responses. Accordingly, characterizing the mechanisms that mediate virulence in pathogenic strains has important public health implications. The *Salmonella* genus includes the two species *Salmonella enterica* and *S*. *bongori*. The former is classified into six subspecies; *S*. *enterica* subspecies *enterica* is the taxon most closely associated with disease^1^.

Recently, multidrug-resistant *S*. *enterica* subspecies *enterica* serotype Typhimurium definitive phage type (DT) 104 has emerged and is now widespread^2–6^. Despite the severity of clinical illness in *S*. Typhimurium DT104 outbreaks suggests that this strain possesses enhanced virulence^3^, no enhanced virulence-associated phenotype has been detected^7^. Within the prophage in the *S*. Typhimurium DT104 genome, we recently identified two genes, *artA* and *artB* (*artAB*), encoding polypeptides with amino acid (a.a.) sequence similarity to the pertussis toxin (Ptx), ADP-ribosyltransferase A subunit (S1 unit), and one of five components of the heteropentameric B subunits (S2 unit)^8^. *S*. Typhimurium DT104 exposure to mitomycin C (MMC), a DNA-damaging agent, induces the expression of prophage-encoded *artAB*. ArtA catalyses ADP-ribosylation of Ptx-sensitive G proteins, and exposure of Chinese hamster ovary (CHO) cells to ArtA/ArtB induces the formation of clusters similar to those observed in Ptx-treated cells^9^. ADP-ribosyltransferase toxins are broadly distributed among highly pathogenic bacteria, and are the primary cause of severe human diseases, such as diphtheria, cholera, and pertussis. All of these toxins belong to the AB subunit class, where the A subunit is the toxic moiety that harbors the active site, and the B subunit is required for receptor binding and the translocation of fragment A across the host cell membrane^10, 11^. Some of these toxins can be structurally classified as AB_5_ toxin family, which are composed of a single A protomer and a pentameric B oligomer^10, 11^.

ArtA/ArtB (ArtAB) is the second reported toxin that catalyses ADP-ribosylation of pertussis toxin-sensitive G proteins; however, the mechanistic basis of its toxicity is unknown. In this study, we demonstrate that in addition to DT104, some non-DT104 *S*. Typhimurium isolates, specifically serotypes *S*. Agoueve and *S*. Worthington, and other *Salmonella* species, *S*. *bongori*, produce ArtAB homologs. Here, we describe the purification of ArtAB toxins from these organisms and the characterization of their biological and physicochemical properties.

## Results

### Distribution of *artAB* in *Salmonella*

To investigate the distribution of *artAB* in *Salmonella* Typhimurium isolates, a panel of 243 DT104 isolates and 302 non-DT104 isolates from our laboratory culture collection was screened by PCR for the presence of *artAB* (see Supplementary Table S1). Of the 243 *S*. Typhimurium DT104 isolates, 237 (97.5%) were positive for *artAB*, while only 11 of the 303 non-DT104 isolates (3.6%) were positive for *artAB*. The nucleotide sequences of *artAB* amplified from 11 isolates — including six non-DT104 isolates — were identical. A panel of 83 other *S*. *enterica* non-Typhimurium serotypes, was screened for presence of *artAB* variants and the genes encoding the pertussis-like toxins A (PltA) and B (PltB), which are homologs of ArtAB and are designated as typhoid toxins^12–14^. Sequences with similarity to *artA*, *artB*, *pltA*, and *pltB* were detected in 19, 18, 19, and 19 of the 83 serotypes, respectively (see Supplementary Table S2). Among the strains harbouring these genes, two *S*. *enterica* serotypes, which are the Agoueve and Worthington, possessed *artA* and *artB* genes with 98% (715/726) and 93% (399/426) sequence identity, respectively, with the corresponding sequences of *S*. Typhimurium DT104. Translated a.a. sequences were 99% identical and 100% similar to *S*. Typhimurium DT104 ArtA, and 83% identical and 99% similar to *S*. Typhimurium DT104 ArtB (see Supplementary Fig. S1). ArtAB homologs were also found in other species of *S*. *bongori*. The a.a. sequence of the *S*. *bongori* strain ATCC43942 ArtA homolog was 95% identical and 99% similar to *S*. Typhimurium DT104 ArtA; however, low conservation was observed between ArtB and the *S*. *bongori* homolog (26% identity and 73% similarity) (see Supplementary Fig. S1).

**Figure 1:**
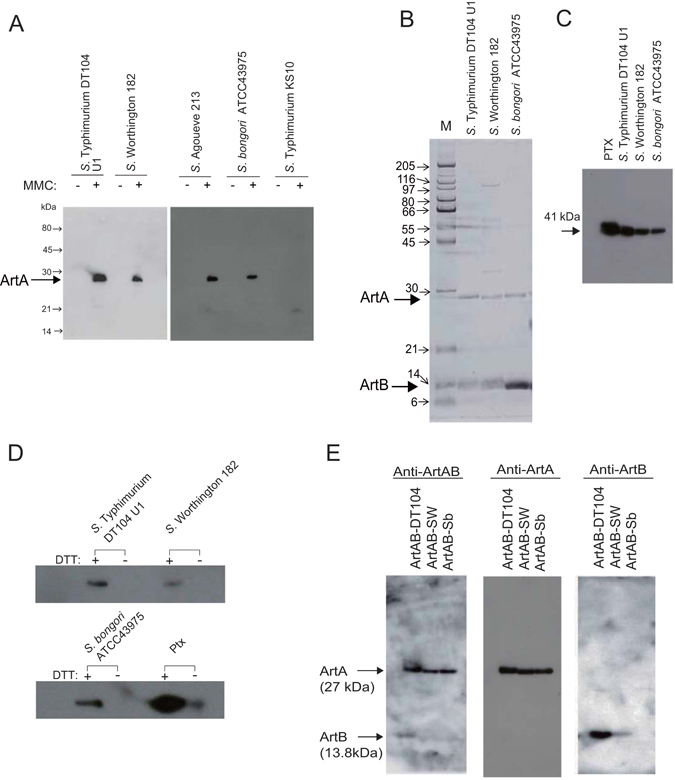
Expression and purification of ArtABs. (**A**) Induction of ArtA expression in *Salmonella* strains by treatment with mitomycin C (MMC). Overnight cultures grown in syncase broth with ( + ) or without (−) 0.5 μg/ml MMC were centrifuged to separate the cells. The supernatant was passed through a 0.22-μm filter, and concentrated 15-fold using a Vivaspin 10 K (GE Healthcare). Total protein contents from the supernatants of *S*. Typhimurium DT104 strain U1, *S*. Worthington strain 182, *S*. Agoueve strain 213, *S*. *bongori* strain ATCC43975, and *S*. Typhimurium strain KST10 were resolved by SDS-PAGE and probed with an antibody against the 14-a.a. peptide corresponding to the Arg^10^–His^23^ sequence of *S*. Typhimurium DT104 ArtA. (**B**) SDS-PAGE analysis of purified ArtABs from *Salmonella* strains. Purified ArtAB from *S*. Typhimurium DT104 stain U1, *S*. Worthington strain 182, and *S*. *bongori* ATCC43975. The gel was stained using a silver staining kit. (**C**,**D**) ADP-ribosylation of Ptx-sensitive G proteins by ArtABs; Ptx-sensitive G proteins from bovine brain (0.1 μg) were incubated with biotinylated NAD and purified ArtABs (100 ng) isolated from the indicated strain for 1 h at 37 °C, and then ADP-ribosylated proteins were analysed by 12.5% SDS-PAGE (**C**), and ADP-ribosylation in the presence or absence of 20 mM DTT (**D**). (**E**) Western blot analysis of purified ArtABs. ArtAB-DT104, ArtAB-SW, and ArtAB-Sb were probed with rabbit anti-ArtAB, anti-ArtA, and anti-ArtB antibodies.

Apart from *S*. Typhimurium strains that carry *artAB* genes, *S*. Worthington, *S*. Agoueve (the other serotype of *S*. *enterica* subspecies *enterica*), and *S*. *bongori* (the other recognized species of *Salmonella*) released a 27-kDa protein into the supernatant of cultures treated with MMC that was recognized by an antibody raised against a 14-a.a. peptide corresponding to the Arg^10^–His^23^ sequence of *S*. Typhimurium DT104 ArtA (Fig. 1A). In the absence of MMC, little or no ArtA was detected in the supernatant of the *Salmonella* cultures (Fig. 1A). *S*. Typhimurium KS10, which lacks *artAB*, did not produce ArtA, even in the presence of MMC (Fig. 1A).

### ArtAB purification

To characterize the products of the pertussis-like toxin genes, ArtABs from bacterial cultures treated with MMC were purified by sequential Affi-Gel Blue, hydroxyapatite, and hydrophobic interaction chromatography using RESOURCE PHE (GE Healthcare, Little Chalfont, UK) (see Supplementary Table S3). Purified ArtAB (58 μg) was obtained from 800 mL of *S*. Typhimurium DT104 culture supernatant, with a final recovery rate of 2.0%. The pertussis-like toxin from *S*. *bongori* did not bind hydroxyapatite under the study conditions; therefore, the hydroxyapatite step was omitted, and instead a Superose 6 Column (GE Healthcare) was used after Affi-Gel Blue chromatography. In sodium dodecyl sulphate polyacrylamide gel electrophoresis (SDS-PAGE), we observed two major bands of 27 and 13.8 kDa, consistent with the expected molecular weights of ArtA and ArtB, respectively (Fig. 1B).

The purified proteins catalysed ADP-ribosylation of the 41-kDa α subunit of the Ptx-sensitive heterotrimeric G protein from bovine brain (Fig. 1C), and this activity was dithiothreitol (DTT)-dependent (Fig. 1D). We designated these toxins from *S*. Typhimurium DT104 strain U1, *S*. Worthington strain 182, and *S*. *bongori* strain ATCC43975 as ArtAB-DT104, ArtAB-SW, and ArtAB-Sb, respectively.

The 27-kDa ArtA proteins from ArtAB-DT104, ArtAB-SW, and ArtAB-Sb were recognized by an antibody raised against ArtA from *S*. Typhimurium DT104 in western blot analysis (Fig. 1E). However, the 13.8-kDa ArtB-SW protein exhibited weak reactivity, and no signal was obtained for ArtB of ArtAB-Sb using an antibody raised against ArtB of ArtAB-DT104 (Fig. 1E).

**Figure 2:**
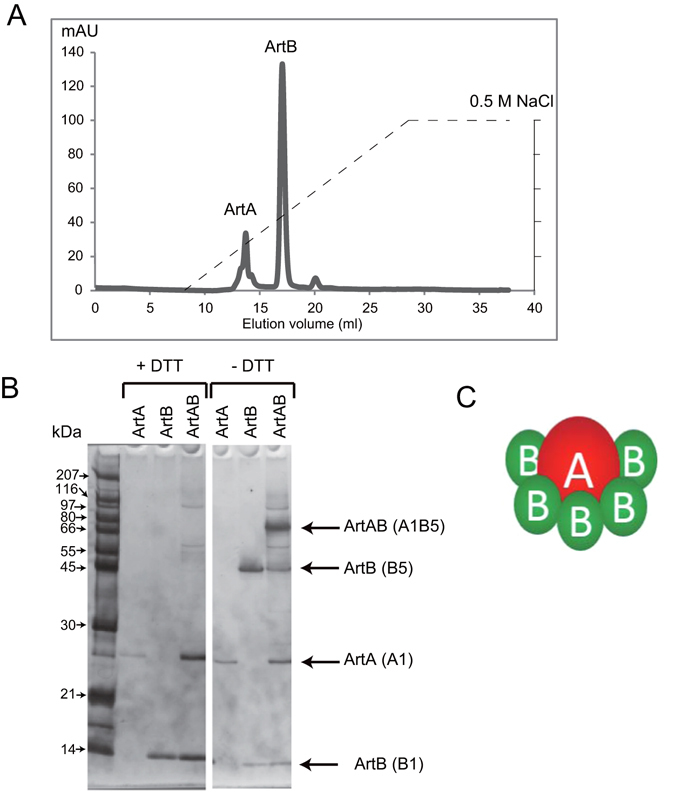
ArtA and pentameric ArtB subunits of ArtAB. (**A**) Separation of ArtA and ArtB by Mono-Q anion exchange chromatography. The dashed line shows the increasing salt gradient (0 to 0.5 M NaCl in 30 mM Tris-HCl, pH 8.8). (**B**) SDS-PAGE analysis of the protein content of the ArtA and ArtB chromatographic fractions obtained by Mono-Q anion exchange chromatography. The gel was stained using a silver staining kit. These along with purified ArtAB were separated by electrophoresis in the presence or absence of 100 mM DTT. Molecular weight standards (kDa) are indicated on the left; positions of the toxin subunits are indicated on the right. (**C**) Schematic illustration of ArtAB subunit structure.

Purified ArtAB-DT104 dissociated into ArtA and ArtB during Mono Q chromatography (Fig. 2A), and the subunits were completely separated. The molar ratio of ArtA to ArtB was approximately 1:5. Electrophoresis of purified ArtAB and the MonoQ fractions of ArtB from DT104 under non-reducing conditions (Fig. 2B) revealed the presence of a high-molecular-mass form of the ArtB pentamer. Furthermore, Coomassie Blue staining of the SDS-PAGE gel revealed that the ArtA and ArtB species from *S*. Typhimurium DT104, *S*. Worthington, and *S*. *bongori* were present at constant molar ratios of 1:5, as determined by densitometry. These results suggest that ArtAB is a member of the AB_5_ toxin family, comprising a single A subunit and pentamer of B subunits (Fig. 2C).

### Biological activity of ArtABs

**Figure 3:**
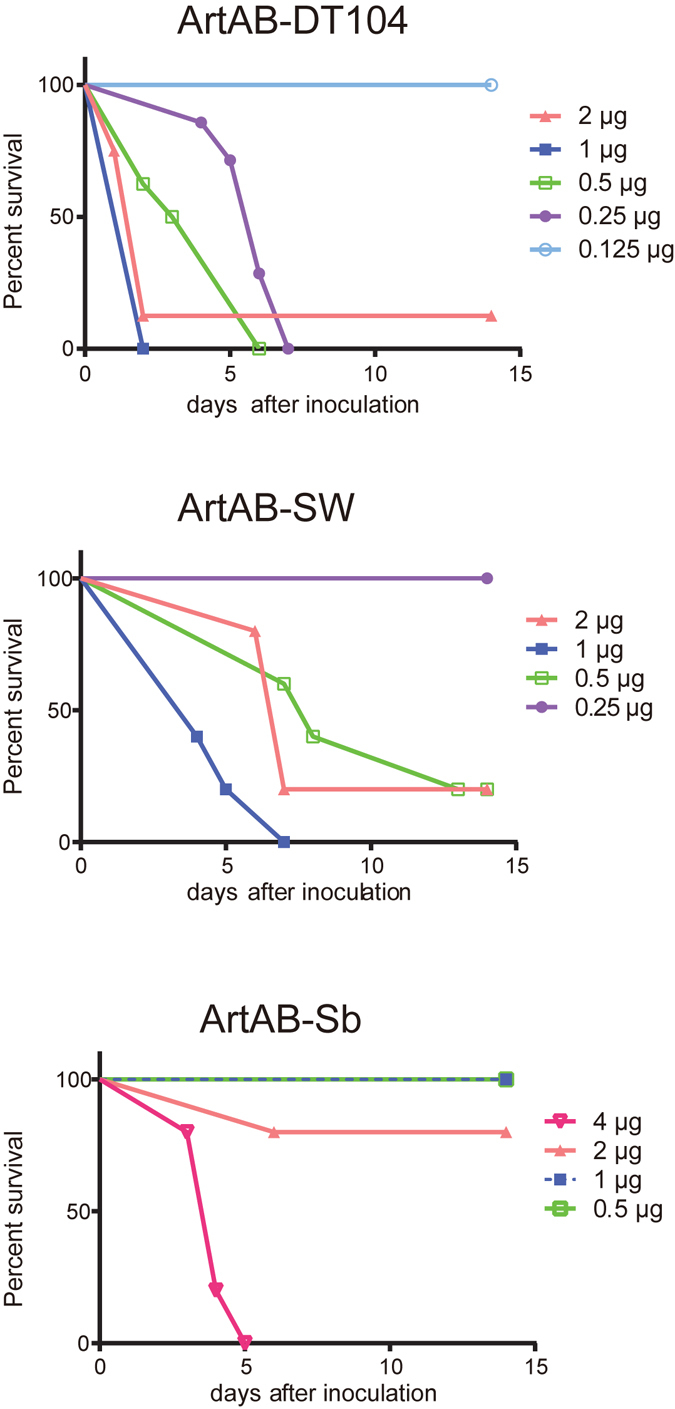
Percentage of surviving mice following challenge with indicated amounts of ArtAB over time. BALB/c mice (n = 5–8) were challenged with graded doses of ArtABs (ArtAB-DT104, ArtAB-SW, or ArtAB-Sb) and observed daily over a 2-week period.

To examine the *in vivo* toxicity of ArtABs, mice (n = 5–8) were administered dilutions of the purified ArtABs by intraperitoneal (i.p.) injection. The 50% lethal dose (LD_50_) of ArtAB-DT104, ArtAB-SW, and ArtAB-Sb in mice was 0.21, 0.44, and 2.75 μg/mouse, respectively (Table 1). Survival time was inversely related to injection dose (Fig. 3). Mice administered 2 μg of ArtAB-DT104, ArtAB-SW, and ArtAB-Sb survived an average of 3.4, 4.0, and 6.0 days, respectively. Five mice injected with 2 μg of heat-treated (at 100 °C for 30 min) ArtAB-DT104 were alive after 14 days. In eight mice that received mixtures of anti-ArtAB-DT104 antibody (100 μg) and a lethal dose of ArtAB (2 μg) by i.p. injection, and the antibody showed a protective effect.

**Table 1:** Lethal activity of ArtAB toxins injected i.p. into BALB/c mice.

Toxin	LD_50_ (μg/mouse)
ArtAB-DT104	0.21
ArtAB-SW	0.44
ArtAB-Sb	2.75

The biological activities of Ptx include stimulation of insulin secretion, induction of leukocytosis, hemagglutinin activity, and induction of CHO cell clustering^15^. Because ArtABs catalyse ADP-ribosylation of Ptx-sensitive G proteins, as shown above, we examined whether ArtABs have the same biological activities as Ptx. Purified ArtAB-DT104 and ArtAB-SW administered at doses of 0.3 and 0.66 μg/mouse, respectively, increased serum insulin levels (P < 0.05); however, an ArtAB-Sb dose of 4.13 μg/mouse failed to increase insulin levels (Table 2). None of the ArtAB toxins promoted leukocytosis in mice, regardless of dose (Table 2). Minimum concentrations of 2, 8, and 0.5 μg/well were required for the hemagglutination (HA) activity of ArtAB-DT104, ArtAB-SW, and Ptx, respectively, and no activity was observed for ArtAB-Sb (Fig. S2A). Furthermore, 100 ng of ArtAB-DT104 and ArtAB-SW induced CHO-K1 cell clustering as Ptx did, which did not occur for 1 μg of ArtAB-Sb (see Supplementary Fig. S2,B).

**Table 2:** Leukocytosis-promoting and islet-activating activity.

Injected toxin	Dose (μg/mouse)	WBC* ± SEM (10^2^/μl)	Insulin secretion (ng/ml) ± SEM
ArtAB-DT104	0.02	27.8 ± 9.75	0.46 ± 0.11
	0.1	18.0 ± 2.12	0.65 ± 0.18
	0.2	25.5 ± 2.60	0.49 ± 0.11
	0.3	21.0 ± 3.70	1.81 ± 0.30**
ArtAB-SW	0.66	17.3 ± 2.82	2.07 ± 0.66**
ArtAB-Sb	4.13	30.7 ± 7.43	0.89 ± 0.20
PBS	—	34.5 ± 8.15	0.39 ± 0.07

*WBC, peripheral white blood cells. **P < 0.05, one-way analysis of variance and Dunnett’s test.

### ArtABs enhance isoproterenol-induced cyclic (c)AMP activation in macrophage-like cells

**Figure 4:**
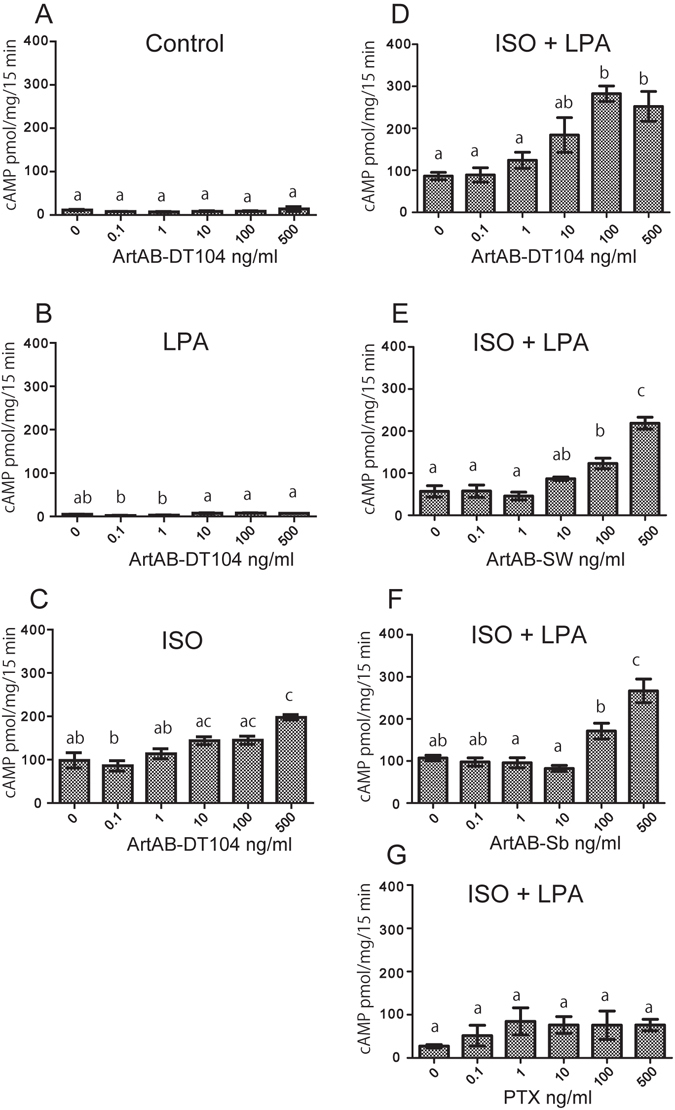
ArtABs induce the upregulation of intracellular cAMP levels in murine macrophage-like cells. (**A**–**G**) cAMP accumulation in RAW 264.7 cells treated with different amounts of ArtAB in the presence of isoproterenol (ISO) and/or LPA. Cells were incubated with ArtABs at the indicated concentrations for 18 h, and then treated with 10 mM ISO and 50 mM LPA at 37 °C for 15 min. Error bars indicate are the standard errors of the means (SEM). Data are from three separate experiments. Values with the same letter are not significantly different (P < 0.05, one-way analysis of variance and Tukey’s test).

Ptx increases basal and humoral adenylate cyclase activity, which results in cAMP accumulation in the host cells. We next examined whether ArtABs could increase cAMP formation, and we observed intracellular cAMP levels within 15 min of treatment with isoproterenol (ISO) in the presence of the phosphodiesterase inhibitor 3-isobutyl-1-methylxanthine (IBMX) in RAW 264.7 murine macrophage-like cells cultured with 0–500 ng of ArtABs. Cells treated with ArtAB without ISO stimulation served as a negative control. Incubation with ISO increased cAMP levels relative to those in unstimulated cells (Fig. 4A,C), and 500 ng/mL ArtAB-DT104 increased cAMP levels relative to the levels in cells treated with control buffer (P < 0.05; Fig. 4C). Lysophosphatidic acid (LPA) did not induce cAMP accumulation (Fig. 4B); however, dose-dependent, ISO-specific ArtAB-DT104-induced cAMP activation was clearly observed in the presence of LPA (Fig. 4D). The cAMP levels observed in the presence of 100 and 500 ng/ml ArtAB-DT104 differed from those observed without ArtAB-DT104 (Fig. 4A and D). Similar results were obtained in the presence of ArtAB-SW and ArtAB-Sb, but not in the presence of Ptx (Fig. 4E–G).

### ADP-ribosylation of RAW 264.7 cell membrane proteins by ArtABs *in vitro* and *in vivo*

**Figure 5:**
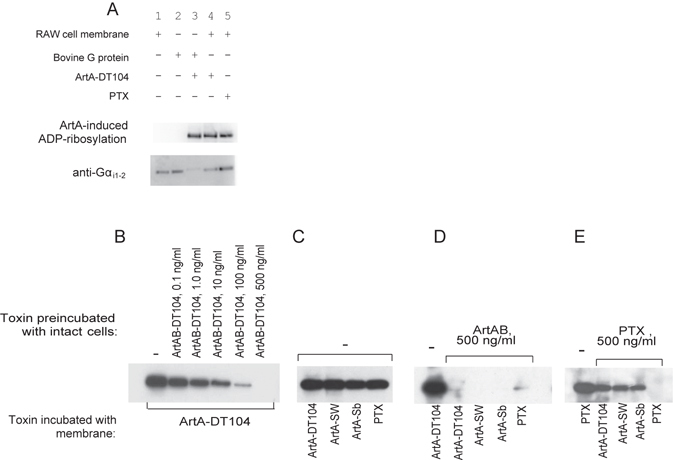
ArtABs catalyse ADP-ribosylation of G proteins in RAW 264.7 cells. (**A**) ADP-ribosylation of G proteins by ArtA-DT104. Upper panel, western blot showing biotin-ADP ribose labelling of Ptx-sensitive G proteins from the bovine brain and RAW 264.7 cell membranes (3.6 μg) by ArtA-DT104 expressed *in vitro* (2 μl) or Ptx (100 ng), in the presence of biotin-NAD. Samples were resolved by 12.5% SDS-PAGE, and ADP-ribosylated proteins were detected by western blotting using peroxidase-conjugated streptavidin as described in Materials and Methods. Lower panel, western blot of the RAW 264.7 cell membrane fraction probed with anti-G_αi1_ and -G_αi2_ IgG antibodies. Lane 1, membrane fraction of RAW 264.7 cells; lane 2, Bovine G proteins; lane 3, Ptx-sensitive G proteins from the bovine brain incubated with ArtA expressed *in vitro*; lane 4, RAW 264.7 cell membranes incubated with *in vitro*-expressed ArtA-DT104; lane 5, RAW 264.7 cell membranes incubated with Ptx. (**B**–**E**) *In vitro* ADP-ribosylation of cell membrane proteins after preincubation of RAW 264.7 cells with ArtAB-DT104 or Ptx. RAW 264.7 cells were incubated with the toxins indicated in upper row (Toxin preincubated with intact cells) for 16 h at 37 °C, and then cell membranes were prepared. The membranes from the pretreated cells were then incubated with the toxins indicated in bottom row (Toxin incubated with membrane; *in vitro*-expressed ArtA or Ptx) in the presence of biotin-NAD. ADP-ribosylated proteins were detected by western blotting as described above. Results are shown for cells preincubated with different concentrations of ArtAB-DT104 (**B**); with no toxin pre-treatment (**C**); with 500 ng of ArtAB-DT104 (**D**); and with 500 ng of Ptx (**E**). In the bottom row, toxin with membrane indicates which toxin was added along with biotinylated NAD to the membrane fractions during the *in vitro* membrane labelling.

ADP-ribosylation of G_i_ protein by Ptx results in cAMP accumulation in cells^16^. Therefore, we examined the ability of the ArtA and ArtAB proteins to promote ADP-ribosylation of G_i_ proteins in RAW 264.7 cell membranes, since their cAMP accumulation was observed by treatment with ArtABs. In the membranes of RAW 264.7 cells, ArtA catalysed the ADP-ribosylation of a 41-kDa protein that was the same size as the α subunit of the Ptx-sensitive heterotrimeric G proteins G_i_, G_o_, and G_t_ from the bovine brain (Fig. 5A). Western blot analysis of the membrane fraction of RAW 264.7 cells and bovine brain G proteins using an antiserum that recognizes the α subunits of heterotrimeric G_i_ proteins (both G_α__i1_ and G_α__i2_) showed that ADP-ribosylated G_α_, which migrated more slowly than non-ADP-ribosylated G_α_ due to the modification contained G_α__i_ (Fig. 5A). These results indicate that the G_αi_ protein is a substrate for ADP-ribosylation by ArtAB *in vitro*. We investigated whether ADP-ribosylation of G proteins occurs in intact RAW 264.7 cells by incubating the cells with various concentrations of ArtAB-DT104, and measuring the decrease in the amount of G protein available for biotin-nicotinamide adenine dinucleotide (NAD) *in vitro*. The membrane fraction containing G proteins was used to detect *in vitro* ADP-ribosylation using ArtA expressed *in vitro*, since ADP-ribosylation is more active using this as compared to purified ArtAB. While untreated cells contained G proteins that can be ADP-ribosylated by ArtA-DT104, incubation with increasing concentrations of ArtAB progressively reduced the amount of available ArtA-DT104-modifiable G protein (Fig. 5B). These results demonstrate that ArtAB-DT104 enters and modifies the G proteins of intact RAW 264.7 cells. Similar results were obtained with ArtAB-SW, ArtAB-Sb, and Ptx (see Supplementary Fig. S3).

Along with Ptx, all the ArtA homologs catalysed ADP-ribosylation of the 41-kDa protein in the RAW 264.7 cell membranes *in vitro* (Fig. 5C); as noted above, one of these proteins is likely G_αi_. To determine whether the target proteins of ArtAB-DT104 are the same as those of ArtAB-Sw, ArtAB-Sb, and Ptx in RAW 264.7 cells, a cellular ADP-ribosylation assay was carried out. RAW 264.7 cells were treated with 500 ng/ml ArtAB-DT104 or Ptx, and their membrane fractions were used for an *in vitro* ADP-ribosylation assay with *in vitro*-translated ArtAs or Ptx. ArtAB-DT104 modified almost all of the available target proteins of ArtA-SW, ArtA-Sb, and Ptx, as evidenced by the absence of a protein band (Fig. 5D). However, preincubation of RAW 264.7 cells with Ptx did not fully block modification of the 41-kDa protein by ArtAs, indicating that ArtA might have ADP-ribosylated additional G_i_-proteins that was not ribosylated by Ptx (Fig. 5E).

## Discussion

We examined the prevalence of *Salmonella* pertussis-like toxins (ArtABs), and found that *artAB* was present in 97.5% (237/243) of *Salmonella* Typhimurium DT104 isolates, but only 3.6% (11/303) of non-DT104 isolates. We also found that two other serotypes, *S*. Worthington and *S*. Agoueve, and an additional species, *S*. *bongori*, that harboured *artAB* homologs. *artAB* is located on a prophage in *S*. Typhimurium DT104, and a number of other toxin genes are encoded by phages; such genes are typically replicated and transcriptionally activated after prophage induction^17, 18^. Shiga toxin (Stx) genes in certain *Escherichia coli* strains and cholera toxin (Ctx) genes in *Vibrio cholera* are located within prophages that are induced by agents that increase toxin production, such as MMC^18^. Although we did not examine the location of *artAB* in non-DT104 *S*. Typhimurium, *S*. Worthington, or *S*. *bongori*, similar to ArtAB-DT104, ArtAB production in these strains was induced by MMC, suggesting that these genes are also encoded by prophages. The pertussis-like toxins ArtAB-DT104, ArtAB-SW, and ArtAB-Sb of *S*. Typhimurium, *S*. Worthington, and *S*. *bongori*, respectively, were purified to homogeneity from MMC-treated culture supernatants, and all three showed potent ADP-ribosyltransferase activity for Ptx-sensitive G proteins. ADP-ribosylation by all three ArtABs was decreased in the absence of DTT, suggesting that ArtA-associated ADP-ribosyltransferase activity depends on thiol reduction, as in the cases of Ctx and Ptx^19–21^.

Ptx belongs to the AB_5_ family of toxins with enzymatic A subunits that disrupt essential host functions and pentameric B subunits that mediate toxin uptake into target cells. AB_5_ toxins also include Ctx, *E*. *coli* heat-labile enterotoxins (LT), Stx, and subtilase cytotoxin (SubAB); the latter is produced by certain Shiga-toxigenic *E*. *coli* strains^11, 22^. The B subunit of Ptx is a heteropentamer comprising four different monomers (S2, S3, two copies of S4, and S5)^21, 23^. ArtAB also belongs to the AB_5_ family; however, ArtAB has a homopentameric B subunit, similar to LT, Ctx, Stx, and SubAB.

We demonstrated that the purified ArtAB is toxic to mice; LD_50_ values were lower for ArtAB-DT104 and ArtAB-SW than ArtAB-Sb. The A subunits of ArtAB-SW and ArtAB-Sb had high sequence identity to that of ArtAB-DT104; all three proteins catalysed ADP-ribosylation of the 41-kDa α-subunits of Ptx-sensitive heterotrimeric G proteins. The B subunits of ArtAB-DT104 and ArtAB-SW were 83% identical, in contrast to the 26% identity between ArtAB-DT104 and ArtAB-Sb. Receptor binding mediated by the B subunit at the cell surface is an essential first step in the intoxication of target cells by AB_5_ toxins. This triggers internalization and intracellular trafficking, allowing the catalytic A subunit to access its substrate. Although the mechanisms underlying the differences in potency among these ArtAB toxins are unclear, B subunit activities, such as receptor recognition or toxin internalization, may play a role. ArtAB-DT104 and ArtAB-SW, but not ArtAB-Sb, induced HA of chicken erythrocytes *in vitro*; in Ptx, this activity is associated with the B oligomer^24^. Furthermore, CHO cells treated with ArtAB-DT104 and ArtAB-SW exhibited clustered growth, similar to cells treated with Ptx. ArtAB-Sb did not have this effect, suggesting that its B subunit may not be recognized by the receptor, and is therefore not internalized by CHO cells.

Ptx catalyses the ADP-ribosylation of the α subunits of the heterotrimeric G_i/o_ protein family, resulting in their inactivation and signal transduction involved in intracellular processes, such as cAMP formation. Ptx exerts pathological effects via cAMP accumulation in host cells^16, 21, 24, 25^. In this study, preincubation of intact RAW 264.7 cells with ArtAB blocked ArtAB-induced ADP-ribosylation of the 41-kDa α subunits of heterotrimeric G_i_ proteins *in vitro*, suggesting that these proteins are substrates for ArtAB toxins *in vivo*. Additionally, incubation of ArtAB-treated RAW 264.7 cells with the β-adrenoreceptor agonist isoproterenol — a specific ligand for G_s_ protein-coupled receptors — leads to dose-dependent stimulation of cAMP production. This effect was enhanced by LPA, an agonist of G_i_ protein-coupled receptors, and inhibits adenylyl cyclase via G_i_^26^. Taken together, although all the targets of ArtA are not known, we showed that ArtAB treatment of cells leads to cAMP accumulation at least via inhibition of G_i_ signalling, similar to Ptx. In a previous study, treatment of G proteins modified by ArtA with HgCl_2_, which cleaves cysteine ADP-ribose bonds, released labelled ADP-ribose, suggesting that ArtA ADP-ribosylates G proteins at a cysteine residue^9^.

The biological effects of Ptx-mediated ADP-ribosylation of heterotrimeric G protein α subunits (G_α__i/o_) include histamine sensitization, stimulation of insulin secretion, and lymphocytosis^24^. All three ArtAB types contain the following three motifs that are conserved in bacterial ADP-ribosylating toxins^27–30^: (i) a putative catalytic glutamate at position 115, (ii) the β/α region with a motif (at positions 50–52) needed for the structural integrity of the NAD-binding site, and (iii) a conserved arginine residue at position 6, which is required for NAD binding in many ARTs (see Supplementary Fig. S1,C). All three ArtAB toxin types catalysed ADP-ribosylation of Ptx-sensitive G proteins. ArtAB-DT104 also induced the activation of insulin secretion; however, this toxin did not exhibit leukocytosis-inducing activity. Two Ptx-sensitive target proteins, G_α__i2_ and G_α__i3_, have been reported in CHO cells, and our previous report revealed differences between ArtA and Ptx with respect to reactivity against these proteins. Additionally, preincubation of RAW 264.7 cells with Ptx did not fully block subsequent ADP-ribosylation of the 41-kDa protein by ArtAB, suggesting that multiple G proteins of this size are substrates for ArtA, but not Ptx. The 41-kDa Ptx substrate in RAW 264.7 cells is a combination of G_αi_ and G_αo_^31^. G_αi_ includes different isoforms of G_α__i1_, G_α__i2_, and G_α__i3_ that have similar molecular weights^32^. Although it is unclear which isoform in RAW 264.7 cells is a substrate for ArtAB, differences in the biological activity of ArtAB and Ptx may be explained by their variable reactivities to target proteins.

Homologs of Ptx have also been identified in *S*. Typhi, and are referred to as pertussis-like toxins A and B (PltA and PltB, respectively)^12–14^. ArtA exhibits 61% identity to PltA for a 224-a.a. segment, while ArtB is 30% identical for a 114-a.a. region. PltA and PltB are required for the delivery of cytolethal distending toxin (Cdt)B from an intracellular compartment into target cells via autocrine and paracrine pathways^12^. CdtB forms a complex with PltA and PltB, which are designated as typhoid toxins. *pltA*, *pltB*, and *cdtB* have also been reported in non-typhoidal *Salmonella* serotypes, such as Montevideo, Schwarzengrund, Bredeney, and Javiana^33, 34^. Therefore, pertussis-like toxins in *Salmonella* can be divided into ArtAB and PltAB types. Although ArtA is homologous to PltA in *S*. Typhi, the target of the ADP-ribosylating activity of PltA is a 100-kDa protein present in the postnuclear supernatant fraction of Henle-470 cells, which is distinct in size from the targets of ArtA and Ptx^12^. Therefore, to our knowledge, ArtA is the only known *Salmonella* enzyme that catalyses ADP-ribosylation of mammalian G proteins.

We previously found that not only MMC but also hydrogen peroxide, an oxidative stressor, induces ArtAB production^9^. Reactive oxygen species generated and released by host cells, such as macrophages or leukocytes, may induce *artAB* expression by intracellular bacteria. Since we do not yet have evidence for ArtAB expression *in vivo*, further studies on the regulation and expression of the toxin are required to clarify the importance of ArtAB in the virulence of *Salmonella*, especially *S*. Typhimurium DT104.

## Methods

### Bacterial strains and animals

The panel of 243 isolates of *S*. Typhimurium DT104, 302 isolates of non-DT104 strains from our laboratory strain culture collection, and NCTC 73 (see Supplementary Table S1) were previously described by Tamamura *et al*.^35^. Apart from serotype Typhimurium, 83 serotypes, including Worthington strain 182 and Agoueve strain 213, as well as *S*. *bongori* strain ATCC43975, are listed in Supplementary Table S2.

Japanese albino rabbits, weighing 1 kg, and 3-week-old female BALB/c mice were purchased from Hokudo Co. (Sapporo, Japan). Animals were maintained and used in accordance with the Guidelines for the Care and Use of Laboratory Animals of the National Institute of Animal Health.

### Cells

CHO cells and the RAW 264.7 murine macrophage cell line were obtained from the American Type Culture Collection (Manassas, VA, USA). CHO cells were cultured in Ham’s F12 medium, and RAW 264.7 cells were cultured in Dulbecco’s Minimal Essential Medium supplemented with 10% foetal calf serum, 0.1 mg/ml streptomycin, and 100 U/ml penicillin G at 37 °C in a humidified atmosphere of 5% CO_2_.

### PCR

*Salmonella* strains were screened for the presence of *artAB* by PCR. DNA fragments corresponding to *artA* and *artB* (*artAB*) were amplified with the primer sets ARTA-1/ARTA-2 (5′-CGTGTTGACTCGAGACC-3′ and 5′-CGTAAACAAGCTCAACTGCTTC-3′) and ARTB-1/ARTB-2 (5′-GGC AAC GTA GGT CCC ATA CA-3′ and 5′-TTG CGT CGT TAT CCA GTG TT-3′. To detect and determine the artAB sequences of S. Typhimurium DT104, DNA fragments of artAB to DT104 were amplified using the primer set ArtAB-DT104-1/ArtAB-DT104-2 (5′-TTA TCC AGC GAG ACG TAA AGT ATA ACT GAG-3′ and 5′-GAC ATA ATC GTC TCA CCA ACA AGA TTC AAA-3′). *pltAB* was also amplified with primer sets PltA1/PltA2 (5′-GTCAGCTCTTTGCCCTCGATG-3′ and 5′-GCGTACTATTCTCGCTCAAC-3′) and PltB1/PltB2 (5′-CCAAAGCATTGTGTCGCACTGC-3′ and 5′-CTCAGACGAAGTTATCAGTG-3′) for *pltA* and *pltB*, respectively. To amplify the *artAB* and *pltAB* of *S*. *bongori*, the primer sets ArtAB-Sb-1/ArtAB-Sb-2 (5′-CTGAGCCATTCGAGCTATCG-3′ and 5′-ATGCCTCCGGGAAACCTCGG-3′) and PltAB-Sb-1/PltAB-Sb-2 (5′-GACTGAATTTTAATGTGGTG-3′ and 5′-CGCACCAATAGGCTATGAAT-3′) were used. PCR products were purified with the QIAquick Gel Extraction Kit (Qiagen, Valencia, CA, USA) according to the manufacturer’s instructions and sequenced on an ABI Prism 377 system (Applied Biosystems, Foster City, CA, USA) using the ABI Prism BigDye Terminator Cycle Sequencing kit (Applied Biosystems). PCR amplification of an internal segment of the 16S-23S spacer region of the bacterial rRNA genes was used to identify DT104, as previously described^36^.

### Purification of ArtAB

*Salmonella* cultures were grown overnight in syncase broth^37^ supplemented with FeCl_3_ to a final concentration of 10 μg/ml. Cultures were diluted 1:40 in 20 ml of syncase broth and grown for 2.5 h at 37 °C on a shaker at 120 rpm; mitomycin C (Sigma, St. Louis, MO, USA) was next added to a final concentration of 0.5 μg/ml, and cultivated for 16 h. Cells were concentrated by centrifugation; the supernatant was passed through 0.22-μm pore filters, and analysed by chromatography. ArtABs (except for ArtAB-Sb) were purified by consecutive elution from Affi-Gel Blue (Bio-Rad, Hercules, CA, USA) and hydroxyapatite columns followed by hydrophobic interaction chromatography (HIC), as previously described for Ptx purification^38^. Desalting or buffer exchange of protein solutions was performed using a PD-10 Column (GE Healthcare).

Culture supernatant filtrate (400 ml) was acidified to pH 6.0 with concentrated HCl before adding 6 ml of Affi-Gel Blue equilibrated with 0.25 M phosphate buffer (pH 6.0). The resultant slurry was stirred at 4 °C for 18 h. The resin was allowed to settle for 1 h and after siphoning off the supernatant solution it was packed into an empty 10-ml column with a Luer lock connection (MoBiTec, Gottingen, Germany) and washed with 0.25 M sodium phosphate (pH 6.0) and then 0.05 M Tris-HCl (pH 7.4), before elution with 0.05 M Tris-HCl (pH 7.4) containing 0.75 M MgCl_2_. Fractions were collected and assayed by western blotting using a rabbit antibody against a 14-a.a. peptide corresponding to the Arg^10^–His^23^ sequence of *S*. Typhimurium DT104 ArtA. Fractions containing ArtAB were pooled, and buffer exchange was performed with 10 mM phosphate buffer (pH 6.0). Proteins were loaded on a 5-ml CHT-I hydroxyapatite column (Bio-Rad) equilibrated with 10 mM phosphate buffer (pH 6.0), washed with 10 mM phosphate buffer (pH 6.0) followed by 0.1 M phosphate buffer (pH 7.0), and eluted with 0.1 M phosphate buffer (pH 7.0) containing 0.5 M NaCl. The buffer in the eluted fraction containing ArtAB was exchanged with 1.5 M ammonium sulphate in 50 mM phosphate buffer (pH 7.0). Then, the proteins were loaded on to a Resource PHE Hydrophobic Interaction Column (GE Healthcare), and eluted with a linear gradient of 1.5 to 0 M ammonium sulphate in 50 mM phosphate buffer solution (pH 7.0) using a fast protein liquid chromatography system (GE Healthcare). The ArtAB fraction was desalted, concentrated in phosphate-buffered saline (PBS), and stored at −80 °C. Since ArtAB from *S*. *bongori* did not bind to the hydroxyapatite gel under these conditions, purification was performed using a Superpose 6 Column (10/300GL; GE Healthcare) with 50 mM phosphate buffer (pH 7.0) and 0.15 M NaCl, after Affi-Gel Blue chromatography, without HIC. For the preparative separation of ArtAB from DT104, the MonoQ Column (GE Healthcare) was loaded with purified ArtAB in a buffer containing 30 mM Tris-HCl (pH 8.8). Proteins were eluted with 20 ml of a linear gradient (0–500 mM) of NaCl in the same sample buffer. Protein concentrations were determined using a Bio-Rad protein assay, with bovine serum albumin as a standard.

### Antibody production

Rabbit antibody against the 14-a.a. peptide corresponding to the Arg^10^–His^23^ sequence of *S*. Typhimurium DT104 ArtA was produced by Sigma Genosys (Ishikari, Japan) as described by Uchida *et al*.^9^. Antibodies against ArtAB, ArtA, and ArtB were obtained from rabbits and mice injected subcutaneously with a 1:1 solution of purified antigen and Titer-Max-Gold (CytRx, Los Angeles, CA, USA) adjuvant three times every 2 weeks. Animals were bled 2 weeks after the last injection. IgG in sera from immunized animals was purified using a HiTrap Protein G HP Column (GE Healthcare) following the manufacturer’s instructions. Anti-G_αi_ rabbit serum was purchased from Calbiochem (San Diego, CA, USA).

### Sandwich enzyme-linked immunosorbent assay (ELISA)

ArtAB in the culture filtrate was measured by sandwich (capture) ELISA. The plate was coated with rabbit anti-ArtAB IgG (10 μg/ml in PBS containing 137 mM NaCl, 2.7 mM KCl, and 10 mM phosphate buffer at 100 μl/well) at 4 °C for 18 h. The plate was blocked with 3% bovine serum albumin in PBS containing 0.02% NaN_3_ (blocking buffer; 400 μl/well) at room temperature for at least 2 h and washed twice with 400 μl of PBS. Culture supernatant was passed through a 0.22-μm membrane filter, and standards were prepared with the blocking buffer; these preparations were added at 50 μl/well, incubated at room temperature for 18 h, and washed four times with PBS (400 μl). Mouse anti-ArtAB IgG (diluted 1:200 in blocking buffer; 100 μl/well) was added and incubated at room temperature for 2 h, then removed by washing three times (400 μl per wash) with PBS. Goat anti-mouse IgG conjugated with horseradish peroxidase (diluted 1:500 in blocking buffer; 100 μl/well) was added and incubated at room temperature for 2 h, followed by three washes (400 μl per wash) with PBS. Tetramethylbenzidine-H_2_O_2_ (TMB Peroxidase EIA Substrate Kit; Bio-Rad) was added, and the reaction was terminated by adding 100 μl of 1 N H_2_SO_4_. The optical density at 450 nm was recorded.

### SDS-PAGE and immunoblotting

Proteins were separated by 12.5% or 15% SDS-PAGE on a 0.1% SDS-Tris-glycine running buffer system and stained with Coomassie blue or silver using a silver staining kit (Daiichi Pure Chemical, Tokyo, Japan). For western blotting, SDS-PAGE gels were transferred to polyvinylidene difluoride (PVDF) membranes (Bio-Rad) in a buffer containing 20% methanol, 25 mM Tris (pH 8.3), and 192 mM glycine. Membranes were incubated for 30 min in blocking buffer consisting of 1% Western Blocking Reagent (Roche Molecular Diagnostics, Indianapolis, IN, USA) in maleic acid buffer (100 mM maleic acid and 150 mM NaCl, adjusted to pH 7.5 with NaOH). The membranes were incubated with rabbit antiserum (1:1000 in maleic acid buffer), and then alkaline phosphatase-conjugated anti-rabbit IgG (Bio-Rad) diluted 1:10,000 in maleic acid buffer. Bands were visualized using a chemiluminescent substrate kit (Bio-Rad).

### *In vitro* translation

*In vitro* transcription/translation of ArtA was performed using Pure System S-S (Post Genome Institute, Tokyo, Japan) as described by Uchida *et al*.^9^. Template DNA was generated by two-step PCR according to the manufacturer’s instructions. Mature ArtA was amplified from chromosomal DNA, and the T7 promoter sequence was amplified. *In vitro* transcription/translation was initiated by adding PCR products from the second step to the Pure System reaction mixture, and incubating at 37 °C for 1 h.

### Cell membrane preparation

To prepare cell membranes, cells grown in 75-cm^2^ flasks were washed twice with PBS, scraped from the flasks, and centrifuged for 5 min at 600 × *g*. Pellets were resuspended in a solution of 0.25 M sucrose, 25 mM Tris-HCl (pH 7.5), and 5 mM MgCl_2_, and homogenized using a sample-grinding kit (GE Healthcare). Homogenates were centrifuged at 600 × *g* for 10 min to remove nuclei and unbroken cells, and the supernatant was centrifuged at 40,000 × *g* for 20 min; the sediment that separated from the supernatant fraction was resuspended in a solution of 20 mM Tris-HCl (pH 7.5) and 5 mM MgCl_2_ and stored at −80 °C.

### ADP-ribosylation assay

ADP-ribosylation of pertussis toxin-sensitive G proteins from the bovine brain (Calbiochem-Novabiochem, San Diego, CA, USA) or cell membrane proteins was determined using biotin-NAD (Trevigen, Gaithersburg, MD, USA), as described by Uchida *et al*.^9^. The *in vitro* ADP-ribosyltransferase reaction mixture (20 μl) contained 0.1 M Tris-HCl (pH 7.6), 0.1 mM ATP, 20 mM DTT, 5 mM thymidine, 10 μM β-NAD, and 0.1 μg of G protein from the bovine brain (Calbiochem-Novabiochem) or 5.6 μg of membrane proteins isolated from RAW 264.7 cells, and either the test substance or Ptx (Biomol, Hamburg, Germany). Ptx was preactivated by incubation in 50 mM Tris-HCl (pH 7.5) containing 50 mM DTT at 37 °C for 20 min. The reaction proceeded for 1 h at 37 °C and was terminated by adding an equal volume of 2× SDS-PAGE sample buffer. Samples were resolved by 12.5% SDS-PAGE. Biotin-ADP-ribosylated proteins were transferred to PVDF membranes, incubated in blocking buffer consisting of 1% Western Blocking Reagent in maleic acid buffer for 30 min, followed by peroxidase-conjugated streptavidin (Vector Laboratories, Burlingame, CA, USA) diluted 1:20,000 in maleic acid buffer for 1 h. Bands were visualized using an enhanced chemiluminescence kit (GE Healthcare) according to the manufacturer’s instructions. ImageJ v.1.6 (National Institutes of Health, Bethesda, MD, USA) was used for the densitometric analysis of band intensity. To assess the efficacy of ADP-ribosylation of G proteins by the toxins *in vivo*, a ‘back ADP-ribosylation’ experiment was performed by pretreating intact cells with toxin, preparing cell membranes from these pretreated cells, and using them in an *in vitro* ADP-ribosylation experiment as described above^39^.

### Mouse lethality

Female BALB/c mice (4 weeks old; n = 5–8) were administered 0.2 ml of sample in graded doses by i.p. injection. Deaths were recorded daily. LD_50_ was calculated as described by Reed^40^. ArtAB neutralization in BALB/c mice was assayed by i.p. injection of 0.2-ml mixtures of 2 μg of ArtAB and 100 μg of rabbit anti-ArtAB IgG preincubated at 37 °C for 18 h. As a control, mice were injected with the same amount of ArtAB incubated with IgG from non-immunized rabbit serum.

### Cell-clustering activity

Clustering of CHO-K1 cells was evaluated as described by Hewlett *et al*.^41^. Briefly, CHO cells grown to confluence in flasks were trypsinized and diluted in F-12 medium with 5% foetal calf serum to a concentration of approximately 2 × 10^4^ cells/ml. A 150-μl volume aliquot of the suspension was added to 8 wells of a flat-bottomed microtiter plate. After allowing 4 h for attachment and stabilization, the test substance (150 μl) was added.

### Hemagglutination (HA) test

The HA test was performed as described elsewhere^15^. Briefly, 50 μl of 0.7% chicken erythrocytes in PBS (v/v) was added to 50 μl of the test substance or Ptx serially diluted with PBS in a microplate. The preparation was incubated at room temperature for 60 min. The minimum amount of sample causing complete agglutination of the red blood cells was recorded.

### Leukocytosis-promoting and islet-activating activities

Assays for both leukocytosis and islet-activating activity have been described elsewhere^15^. Briefly, 5-week-old female BALB/c mice (n = 4–10) were administered the test substance by i.p. injection. On day 3, tail vein blood samples were drawn and leukocytes were counted. To measure islet-activating activity, serum insulin in day 3 blood samples was measured 15 min after injection of 50% glucose (0.5 ml) using an ELISA kit (Morinaga Institute of Biological Science, Yokohama, Japan) according to the manufacturer’s instructions.

### Toxin treatment and cAMP assessment

RAW 264.7 cells were harvested and subcultured in a 24-well plate at a density of 1.0 × 10^5^ cells/well. After 18 h, the medium was replaced with 0.5 ml of fresh medium containing toxins. Cultures were incubated for an additional 24 h and washed twice with 1 ml of buffered saline solution containing 137 mM NaCl, 5 mM KCl, 5.6 mM glucose, 1 mM EGTA, and 5 mM HEPES (pH 7.4). After washing, 0.4 ml of buffered saline supplemented with 1 mM MgCl_2_ and 1 mM IBMX was added to the cultures, which were incubated at 37 °C for 30 min; this incubation was followed by a 15-min incubation at 37 °C with 10 μM isoproterenol or 10 μM forskolin and/or 50 μM LPA. The medium was next removed by aspiration and cells were washed with PBS. We next added 0.4 ml of lysis reagent from the cAMP Enzyme Immunoassay System Kit (GE Healthcare). The cAMP assay was performed according to the manufacturer’s instructions.

### Statistical analysis

Differences were evaluated using one-way ANOVA with Tukey’s test or Dunnett’s test. All P values were calculated using GraphPad Prism version 6.0 and were interpreted as significant at values less than 0.05.

### Study approval

All animal procedures were carried out in strict accordance with local guidelines and with ethical approval from the National Institute of Animal Health.

## Electronic supplementary material

Table S1–S3

Figure S1–S3

## References

[1] Guibourdenche M (2010). Supplement 2003–2007 (No. 47) to the White-Kauffmann-Le Minor scheme. Res. Microbiol..

[2] Threlfall EJ, Frost JA, Ward LR, Rowe B (1994). Epidemic in cattle and humans of *Salmonella* typhimurium DT 104 with chromosomally integrated multiple drug resistance. Vet. Rec..

[3] Glynn MK (1998). Emergence of multidrug-resistant *Salmonella enterica* serotype Typhimurium DT104 infections in the United States. N. Engl. J. Med..

[4] Villar RG (1999). Investigation of multidrug-resistant *Salmonella* serotype Typhimurium DT104 infections linked to raw-milk cheese in Washington State. JAMA.

[5] Sameshima T (2000). *Salmonella* Typhimurium DT104 from livestock in Japan. Jpn. J. Infect. Dis..

[6] Leekitcharoenphon P (2016). Global Genomic Epidemiology of *Salmonella enterica* Serovar Typhimurium DT104. Appl. Environ. Microbiol..

[7] Allen CA (2001). *In vitro* and *in vivo* assessment of *Salmonella enterica* serovar Typhimurium DT104 virulence. Infect. Immun..

[8] Saitoh M (2005). The *artAB* genes encode a putative ADP-ribosyltransferase toxin homologue associated with *Salmonella enterica* serovar Typhimurium DT104. Microbiology.

[9] Uchida I (2009). *Salmonella enterica* serotype Typhimurium DT104 ArtA-dependent modification of pertussis toxin-sensitive G proteins in the presence of [32P]NAD. Microbiology.

[10] Burnette WN (1994). AB5 ADP-ribosylating toxins: comparative anatomy and physiology. Structure.

[11] Merritt EA, Hol WG (1995). AB5 toxins. Curr. Opin. Struct. Biol..

[12] Spano S, Ugalde JE, Galan JE (2008). Delivery of a *Salmonella* Typhi exotoxin from a host intracellular compartment. Cell Host Microbe.

[13] Song J, Gao X, Galan JE (2013). Structure and function of the *Salmonella* Typhi chimaeric A(2)B(5) typhoid toxin. Nature.

[14] Galan JE (2016). Typhoid toxin provides a window into typhoid fever and the biology of *Salmonella* Typhi. Proc. Natl. Acad. Sci. USA.

[15] Sato H, Ito A, Chiba J, Sato Y (1984). Monoclonal antibody against pertussis toxin: effect on toxin activity and pertussis infections. Infect. Immun..

[16] Katada T, Ui M (1982). Direct modification of the membrane adenylate cyclase system by islet-activating protein due to ADP-ribosylation of a membrane protein. Proc. Natl. Acad. Sci. USA.

[17] Wagner PL, Acheson DW, Waldor MK (2001). Human neutrophils and their products induce Shiga toxin production by enterohemorrhagic *Escherichia coli*. Infect. Immun..

[18] Wagner PL, Waldor MK (2002). Bacteriophage control of bacterial virulence. Infect. Immun..

[19] Moss J (1983). Activation by thiol of the latent NAD glycohydrolase and ADP-ribosyltransferase activities of *Bordetella pertussis* toxin (islet-activating protein). J. Biol. Chem..

[20] Mekalanos JJ, Collier RJ, Romig WR (1979). Enzymic activity of cholera toxin. I. New method of assay and the mechanism of ADP-ribosyl transfer. J. Biol. Chem..

[21] Locht C, Coutte L, Mielcarek N (2011). The ins and outs of pertussis toxin. FEBS J..

[22] Paton AW, Srimanote P, Talbot UM, Wang H, Paton JC (2004). A new family of potent AB(5) cytotoxins produced by Shiga toxigenic *Escherichia coli*. J. Exp. Med..

[23] Krueger KM, Barbieri JT (1995). The family of bacterial ADP-ribosylating exotoxins. Clin. Microbiol. Rev..

[24] Mangmool S, Kurose HG (2011). (i/o) protein-dependent and -independent actions of Pertussis Toxin (PTX). Toxins.

[25] Locht C (1999). Molecular aspects of *Bordetella pertussis* pathogenesis. Int. Microbiol..

[26] Moolenaar WH (1995). Lysophosphatidic acid, a multifunctional phospholipid messenger. J. Biol. Chem..

[27] Carroll SF, Collier RJ (1984). NAD binding site of diphtheria toxin: identification of a residue within the nicotinamide subsite by photochemical modification with NAD. Proc. Natl. Acad. Sci. USA.

[28] Domenighini M, Rappuoli R (1996). Three conserved consensus sequences identify the NAD-binding site of ADP-ribosylating enzymes, expressed by eukaryotes, bacteria and T-even bacteriophages. Mol. Microbiol..

[29] Collier RJ (2001). Understanding the mode of action of diphtheria toxin: a perspective on progress during the 20th century. Toxicon.

[30] Pallen, M. J., Lam, A. C., Loman, N. J. & McBride, A. An abundance of bacterial ADP-ribosyltransferases-implications for the origin of exotoxins and their human homologues. *Trends*. *Microbiol*. **9**, 302–307; discussion 308 (2001). 10.1016/s0966-842x(01)02074-1 11435081

[31] Burch RM, Jelsema C, Axelrod J (1988). Cholera toxin and pertussis toxin stimulate prostaglandin E2 synthesis in a murine macrophage cell line. J. Pharmacol. Exp. Ther..

[32] Backlund PS (1988). Immunochemical and electrophoretic characterization of the major pertussis toxin substrate of the RAW264 macrophage cell line. Biochemistry.

[33] den Bakker HC (2011). Genome sequencing reveals diversification of virulence factor content and possible host adaptation in distinct subpopulations of *Salmonella enterica*. BMC Genomics.

[34] Mezal EH, Bae D, Khan AA (2014). Detection and functionality of the CdtB, PltA, and PltB from *Salmonella enterica* serovar Javiana. Pathog. Dis..

[35] Tamamura Y (2011). Molecular epidemiology of *Salmonella enterica* serovar Typhimurium isolates from cattle in Hokkaido, Japan: evidence of clonal replacement and characterization of the disseminated clone. Appl. Environ. Microbiol..

[36] Pritchett LC, Konkel ME, Gay JM, Besser TE (2000). Identification of DT104 and U302 phage types among *Salmonella enterica* serotype Typhimurium isolates by PCR. J. Clin. Microbiol..

[37] Finkelstein RA, Atthasampunna P, Chulasamaya M, Charunmethee P (1966). Pathogenesis of experimental cholera: biologic activities of purified procholeragen A. J. Immunol..

[38] Svoboda M, Hannecart-Pokorni E, Borremans M, Christophe J (1986). Rapid purification of *Bordetella pertussis* toxin by alternating affinity and hydrophobic chromatography. Anal. Biochem..

[39] Ribas C, Takesono A, Sato M, Hildebrandt JD, Lanier SM (2002). Pertussis toxin-insensitive activation of the heterotrimeric G-proteins Gi/Go by the NG108-15 G-protein activator. J. Biol. Chem..

[40] Reed, L. J. & Muench, H. A simple method of estimating fifty per cent endpoints. *Am*. *J*. *Hyg*. 493–497 (1938).

[41] Hewlett EL, Sauer KT, Myers GA, Cowell JL, Guerrant RL (1983). Induction of a novel morphological response in Chinese hamster ovary cells by pertussis toxin. Infect. Immun..

