# Supplementary material for: Characterization of pertussis-like toxin from Salmonella spp. that catalyzes ADP-ribosylation of G proteins
Source: Sci Rep. 2017 Jun 1;7:2653. doi: 10.1038/s41598-017-02517-2 (PMC5454059; doi:10.1038/s41598-017-02517-2)
Supplement: Supplementary file 2 — Figure S1–S3 [file 41598_2017_2517_MOESM2_ESM.pdf]

Characterization of pertussis-like toxin from *Salmonella* spp. that catalyzes ADP-ribosylation of G proteins

Running title: ADP-ribosyltransferase toxin of *Salmonella*

Yukino Tamamura[1], Kiyoshi Tanaka[2], and Ikuo Uchida [3*]

[1]Bacterial and Parasitic Diseases Research Division, National Institute of Animal Health, Tsukuba, Ibaraki, 305-0856, Japan

[2]Dairy Hygiene Research Division, Hokkaido Research Station, National Institute of Animal Health, 4 Hitsujigaoka, Toyohira, Sapporo 062-0045, Japan

[3]Veterinary Bacteriology, Department of Pathology, School of Veterinary Medicine, Rakuno Gakuen University, 582 Bunkyoudai-midorimachi, Ebetsu, Hokkaido, 069-8501, Japan

*Corresponding author:
Ikuo UCHIDA
Veterinary Bacteriology,
Department of Pathology, School of Veterinary Medicine,
Rakuno Gakuen University,
582 Bunkyoudai-midorimachi, Ebetsu, Hokkaido, 069-8501, Japan
Telephone: (81)-11-388-4899
E-mail: i-uchida@rakuno.ac.jp

S

A

```
S. bongori  ATCC43975          1 VDFVYRVDSRPPDVIFRDGFSSHGNNRNLQQHIRGDSCAAGSRDSNYIATTSDINETYNI
S. Typhimurium DT104 U1        1 VDFVYRVDSRPPDVIFRDGFNSHGNNRNLQQHIRGDSCSAGSRDSNYIATTSDINETYNI
S. Worthington 182             1 VDFVYRVDSRPPDVIFRDGFSSHGNNRNLQQHIRGDSCSAGSRDSNYIATTSDINETYNI
S. Agoueve 213                 1 VDFVYRVDSRPPDVIFRDGFSSHGNNRNLQQHIRGDSCSAGSRDSNYIATTSDINETYNI

S. bongori  ATCC43975         61 ARVYYSRATFSGRLYRYRIRADNSFYSLPPSVAYIESRGVQFNHFERVMMRLQSEYVAVN
S. Typhimurium DT104 U1       61 ARVYYSRTTFSGRLYRYRIRADNSFYSLPPSVAYIESRGIQFSHFERVMMRLQSEYVAVN
S. Worthington 182            61 ARVYYSRTTFSGRLYRYRIRADNSFYSLPPSVAYIESRGIQFSHFERVMMRLQSEYVAVN
S. Agoueve 213                61 ARVYYSRTTFSGRLYRYRIRADNSFYSLPPSVAYIESRGIQFSHFERVMMRLQSEYVAVN

S. bongori  ATCC43975        121 SIPIENIQEAVELVYDRNTSQVRDGPGTSNSRYLRVSTQSNPGVIPNLPVPQVSTRERIS
S. Typhimurium DT104 U1      121 SIPIENIQEAVELVYDRNTSQVRDGSGTSNSRYLRVSTQSNPGVIPNLPVPQVSTRERIS
S. Worthington 182           121 SIPIENIQEAVELVYDRNTSQVRDGSGTSNSRYLRVSTQSNPGVIPNLPVPQVSTRERIS
S. Agoueve 213               121 SIPIENIQEAVELVYDRNTSQVRDGSGTSNSRYLRVSTQSNPGVIPNLPVPQVSTRERIS

S. bongori  ATCC43975        181 AFGTLISACFSMRGVRRDDTRINSNYYEMEFYDARGVLTELLK
S. Typhimurium DT104 U1      181 AFGTLISACFSMRGVRRDDARSNYNYYEMEFYDARGVLTELLD
S. Worthington 182           181 AFGTLISACFSMRGVRRDDARSNYNYYEMEFYDARGVLTELLN
S. Agoueve 213               181 AFGTLISACFSMRGVRRDDARSNYNYYEMEFYDARGVLTELLN
```

B

```
S. bongori  ATCC43975          1 SNVYATVNNWYLKDTTKYENVKITNVFYAPYLHSPRICAYFTASS-GGSNV----TGCAV
S. Typhimurium DT104 U1        1 -----------ADYNTYQSNVQINNLSYGVYRSGDKESQFFCVGLKRGSQVPNVHTICKI
S. Worthington 182             1 -----------ADYNTYQSNVQINNLSHGVYKSGGKDSQFFCIGLNNESQIPNANTMCKM
S. Agoueve 213                 1 -----------ADYNTYQSNVQINNLSHGVYKSGGKDSQFFCIGLNNESQIPNANTMCKM

S. bongori  ATCC43975         56 ADNGYYQKNAGQTSPFMEIFDTVKYFYTTGEKISVYIRINAFS--HFDSSVSQNEIVAIG
S. Typhimurium DT104 U1       50 DV------FGTHKQGFDNMLATARYYYATGEDVRIYYKENVWTDRNFTAAFSGNELIAIT
S. Worthington 182            50 DV------FGTHKQGFDNMLATARYYYTTGEKVRIYYKENVWADRNFTAGFSGNELIAIT
S. Agoueve 213                50 DV------FGTHKQGFDNMLATARYYYTTGEKVRIYYKENVWADRNFTAGFSGNELIAIT

S. bongori  ATCC43975        114 TCN--QWCFGEIIK-
S. Typhimurium DT104 U1      104 TCTSSDYCMGPTLPN
S. Worthington 182           104 TCSSIDYCMGPTLPN
S. Agoueve 213               104 TCSSIDYCMGPTLPN
```

C

		Necessary for NAD binding			NAD-binding site			Catalytic glutamate
ArtA-DT104	2	DFVY**R**VDSR	…	45	SNYIA**TTS**DINE	…	109	MMRLQS**E**YVAL
ArtA-SW	2	DFVY**R**VDSR	…	45	SNYIA**TTS**DINE	…	109	MMRLQS**E**YVAL
ArtA-Sb	2	DFVY**R**VDSR	…	45	SNYIA**TTS**DINE	…	109	MMRLQS**E**YVAL
PltA	1	DFVY**R**VDST	…	35	SRYIA**TTS**SVNQ	…	93	MMRLQR**E**YVST
MPN372	6	RFVY**R**VDLR	…	44	RSYFI**STS**ETPT	…	126	SFAYQR**E**WFTD
Ptx	5	PATV**R**YDSR	…	46	NSAFV**STS**SSRR	…	122	LATYQS**E**YLAH
Ltx	3	DRLY**R**ADSR	…	56	DDGYV**STS**ISLR	…	106	PHPYEQ**E**VSAL
Ctx	3	DKLY**R**YVYE	…	56	DDGYV**STS**ISLR	…	106	PHDPEQ**E**VSAL

Figure S1. Alignment of the amino acid (a.a.) sequences of ArtA (A) and ArtB (B) from *Salmonella* Typhimurium DT104 U1, *S*. Agoueve 213, *S*. Worthington 182, and *S. bongori* ATCC43975. The alignment was generated with ClustalW (http://www.ebi.ac.uk/clustalw/), and sequences were shaded using BoxShade (http://www.ch.embnet.org/software/BOX_form.html). Identical and similar regions are indicated by black and grey boxes, respectively. (C) Alignment of the residues conserved between ArtA and other ADP-ribosyltransferase toxins. PltA, *S*. Typhi pertussis-like toxin A; MPN372, *Mycoplasma pneumonia* toxin; Ptx, pertussis toxin; Ltx, *Escherichia coli* heat-labile enterotoxin; Ctx, cholera toxin. Conserved residues that are critical for Ptx function are shown in bold.

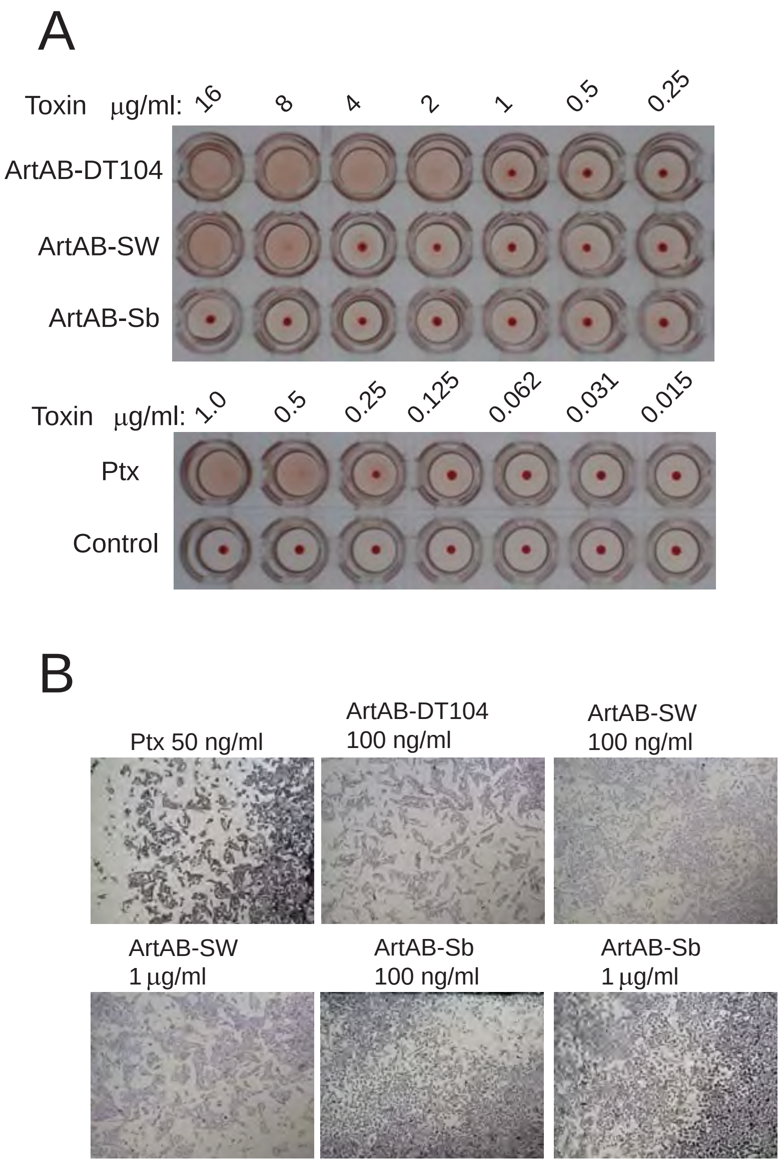

Figure S2. Hemagglutinin (HA) and CHO cell clustering activity of ArtABs and Ptx. (A) HA activity of ArtABs and Ptx. HA activity at different concentrations of sample (serial 2-fold dilutions) using chicken erythrocytes. (B) CHO cell clustering induced by ArtABs. Cells were exposed to Ptx (50 ng/well), ArtAB-DT104 (100 ng/well), ArtAB-SW (1 μg and 100 ng/well), or ArtAB-Sb (1 μg and 100 ng/well).

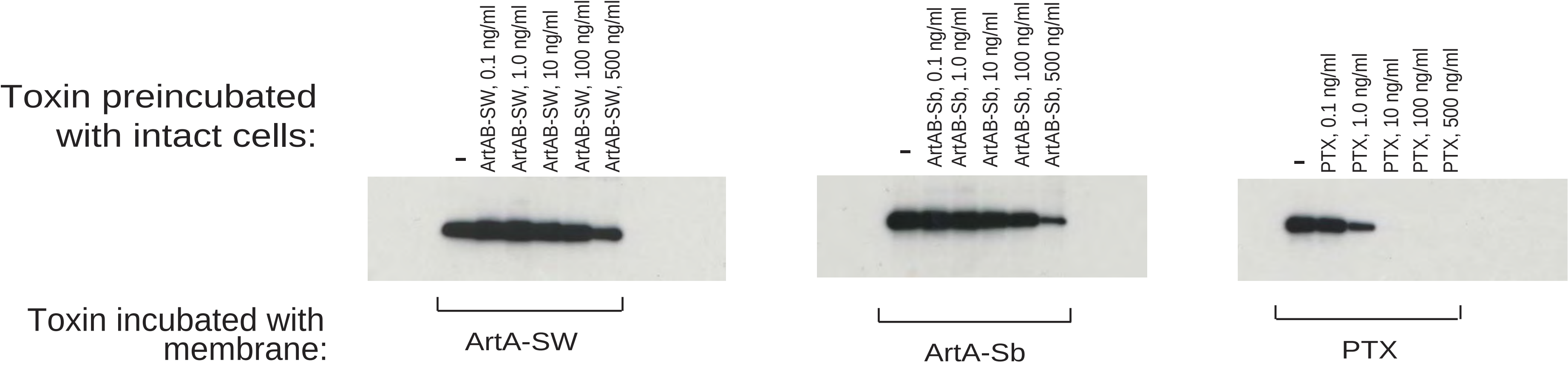

Figure S3. In vitro ADP-ribosylation of cell membrane proteins after pre-treatment of RAW 264.7 cells with ArtAB-SW, ArtAB-Sb, or PTX. RAW 264.7 cells were incubated with various concentrations of ArtAB-SW, ArtAB-Sb, or PTX, indicated in upper row; membranes were subsequently prepared. Membranes prepared from these pretreated cells were incubated with the in vitro expressed toxin indicated in bottom row ("Toxin with membrane"), along with biotinylated NAD, for an in vitro membrane labelling experiment. Samples were resolved by 12.5% SDS-PAGE, and ADP-ribosylated proteins were detected by western blotting using peroxidase-conjugated streptavidin, as described in the Materials and Methods.

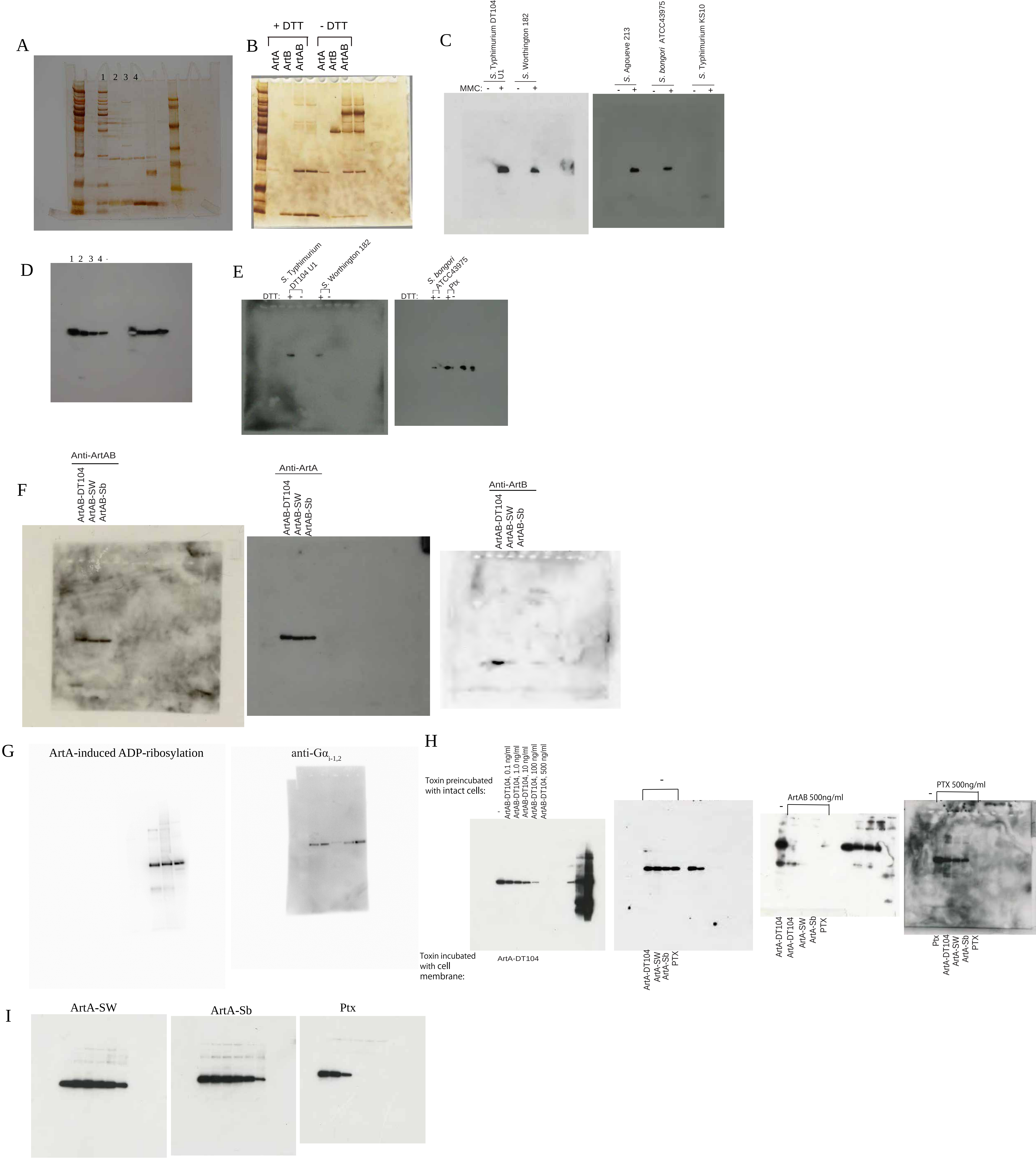

Figure S4. Full length gels and membrane. (A) Full length gel for Figure 1B. 1: Marker 2: *S.* Typhimurium DT104 U1 3: *S.* Worthington 182 4: *S.bongori* ATCC43975
(B) Full length gel for Figure 2B. (C) Full length membrane for Figure 1A. (D) Full length membrane for Figure 1C. 1: PTX 2:*S.* Typhimurium DT104 U1 3: *S.* Worthington 182 4: *S.bongori* ATCC43975
(E) Full length membrane for Figure1D. (F) Full length membrane for Figure 1E. (G) Full length membrane for Figure 5A. (H) Full length membrane for Figure 5B-E.
(I) Full length membrane for Fig.S3.
